# miR-509-3p is clinically significant and strongly attenuates cellular migration and multi-cellular spheroids in ovarian cancer

**DOI:** 10.18632/oncotarget.8412

**Published:** 2016-03-27

**Authors:** Yinghong Pan, Gordon Robertson, Lykke Pedersen, Emilia Lim, Anadulce Hernandez-Herrera, Amy C. Rowat, Sagar L. Patil, Clara K. Chan, Yunfei Wen, Xinna Zhang, Upal Basu-Roy, Alka Mansukhani, Andy Chu, Payal Sipahimalani, Reanne Bowlby, Denise Brooks, Nina Thiessen, Cristian Coarfa, Yussanne Ma, Richard A. Moore, Jacquie E. Schein, Andrew J. Mungall, Jinsong Liu, Chad V. Pecot, Anil K. Sood, Steven J.M. Jones, Marco A. Marra, Preethi H. Gunaratne

**Affiliations:** ^1^ Department of Biochemistry and Biology, University of Houston, Houston, TX, USA; ^2^ Canada's Michael Smith Genome Sciences Centre, BC Cancer Agency, Vancouver, BC, Canada; ^3^ Department of Biology, University of Copenhagen, Copenhagen, Denmark; ^4^ Department of Integrative Biology and Physiology, University of California, Los Angeles, CA, USA; ^5^ Department of Gynecologic Oncology and Reproductive Medicine, The University of Texas MD Anderson Cancer Center, Houston, TX, USA; ^6^ Center for RNA Interference and Non-Coding RNA, The University of Texas MD Anderson Cancer Center, Houston, TX, USA; ^7^ Department of Cancer Biology, The University of Texas MD Anderson Cancer Center, Houston, TX, USA; ^8^ Department of Pathology, The University of Texas MD Anderson Cancer Center, Houston, TX, USA; ^9^ Department of Microbiology, New York University School of Medicine, New York, NY, USA; ^10^ UNC Lineberger Comprehensive Cancer Center, Thoracic Medical Oncology, University of North Carolina, Chapel Hill, NC, USA; ^11^ Department of Pathology and Immunology, Baylor College of Medicine, Houston, TX, USA; ^12^ Department of Molecular Biology and Biochemistry, Simon Fraser University, Burnaby, BC, Canada; ^13^ Department of Medical Genetics, University of British Columbia, Vancouver, BC, Canada; ^14^ Department of Molecular and Cellular Biology, Baylor College of Medicine, Houston, TX, USA

**Keywords:** microRNA 509-3p, ovarian cancer, extracellular matrix (ECM), YAP1, spheroid formation

## Abstract

Ovarian cancer presents as an aggressive, advanced stage cancer with widespread metastases that depend primarily on multicellular spheroids in the peritoneal fluid. To identify new druggable pathways related to metastatic progression and spheroid formation, we integrated microRNA and mRNA sequencing data from 293 tumors from The Cancer Genome Atlas (TCGA) ovarian cancer cohort. We identified miR-509-3p as a clinically significant microRNA that is more abundant in patients with favorable survival in both the TCGA cohort (*P* = 2.3E–3), and, by *in situ* hybridization (ISH), in an independent cohort of 157 tumors (*P* < 1.0E–3). We found that miR-509-3p attenuated migration and disrupted multi-cellular spheroids in HEYA8, OVCAR8, SKOV3, OVCAR3, OVCAR4 and OVCAR5 cell lines. Consistent with disrupted spheroid formation, in TCGA data miR-509-3p's most strongly anti-correlated predicted targets were enriched in components of the extracellular matrix (ECM). We validated the Hippo pathway effector *YAP1* as a direct miR-509-3p target. We showed that siRNA to *YAP1* replicated 90% of miR-509-3p-mediated migration attenuation in OVCAR8, which contained high levels of YAP1 protein, but not in the other cell lines, in which levels of this protein were moderate to low. Our data suggest that the miR-509-3p/*YAP1* axis may be a new druggable target in cancers with high *YAP1*, and we propose that therapeutically targeting the miR-509-3p/*YAP1*/ECM axis may disrupt early steps in multi-cellular spheroid formation, and so inhibit metastasis in epithelial ovarian cancer and potentially in other cancers.

## INTRODUCTION

Epithelial ovarian cancer (EOC) is the most lethal gynecological malignancy, typically presenting as an aggressive, advanced stage cancer with widespread metastases in the peritoneum [1]. It metastasizes primarily by cells exfoliating into the peritoneal fluid, and aggregating into multicellular spheroids that invade the peritoneal membrane [2, 3] and can resist chemotherapy [4–6]. While patients typically respond initially to cytoreductive surgery and to platinum- and taxane-based chemotherapy, most develop relapsed disease that becomes refractory to all current therapies. There is a pressing need for new therapeutic strategies that sensitize these cancers to current therapies.

Recently, The Cancer Genome Atlas (TCGA) Research Network characterized 489 high-grade serous ovarian cancer (HGSOC) tumors [7], using microarrays for gene and microRNA expression. Here, we report results from 293 TCGA ovarian tumors, using messenger RNA (mRNA) and microRNA (miRNA/miR) sequencing data. miRNAs are ~22-nucleotide non-coding RNAs that regulate transcript stability and translation, primarily by binding sites in 3′-UTRs [8]. We anticipated that the sequencing data could yield new insights by offering a wider dynamic range than microarrays, higher spatial resolution and sensitivity, isoform-specific mRNA expression, and better discrimination between miRNA stem-loops and mature miRNA strands [9].

We identified the X-linked miR-509-3p as a clinically significant miRNA. The miR was more abundant in patients with favorable survival in both the TCGA and an independent cohort. In attenuated migration, invasion, and aggregation into three-dimensional (3D) spheroids in the six ovarian cancer cell lines tested: HEYA8, OVCAR8, SKOV3, OVCAR3, OVCAR4 and OVCAR5. We showed that YAP1, a key oncogene in a subset of ovarian and other cancers, is a direct downstream target of miR-509-3p. We also found that siRNA to *YAP1* was necessary and sufficient to replicate ~90% of miR-509-3p-mediated attenuation of migration in OVCAR8, which contained high levels of YAP1 protein. However, siYAP1 had no impact on migration attenuation or multi-cellular spheroids in the other cell lines, suggesting that the miR-509-3p/*YAP1* axis is relevant only in cells containing high *YAP1*.

Since miR-509-3p's strongest anti-correlated predicted targets in TCGA ovarian tumors were enriched for components of the extracellular matrix (ECM), and effects on ECM components are consistent with effects on 3D spheroid formation, we propose that miR-509-3p may act in ovarian cancers by targeting both ECM components and *YAP1*. We anticipate that the miR-509-3p/*YAP1*/ECM axis offers a novel therapeutic focus for controlling metastatic progression in HGSOC.

## RESULTS

### An independent component that discriminates overall survival is enriched in Xq27.3 miR cluster members, and in ECM and cell adhesion genes

**Figure 1:**
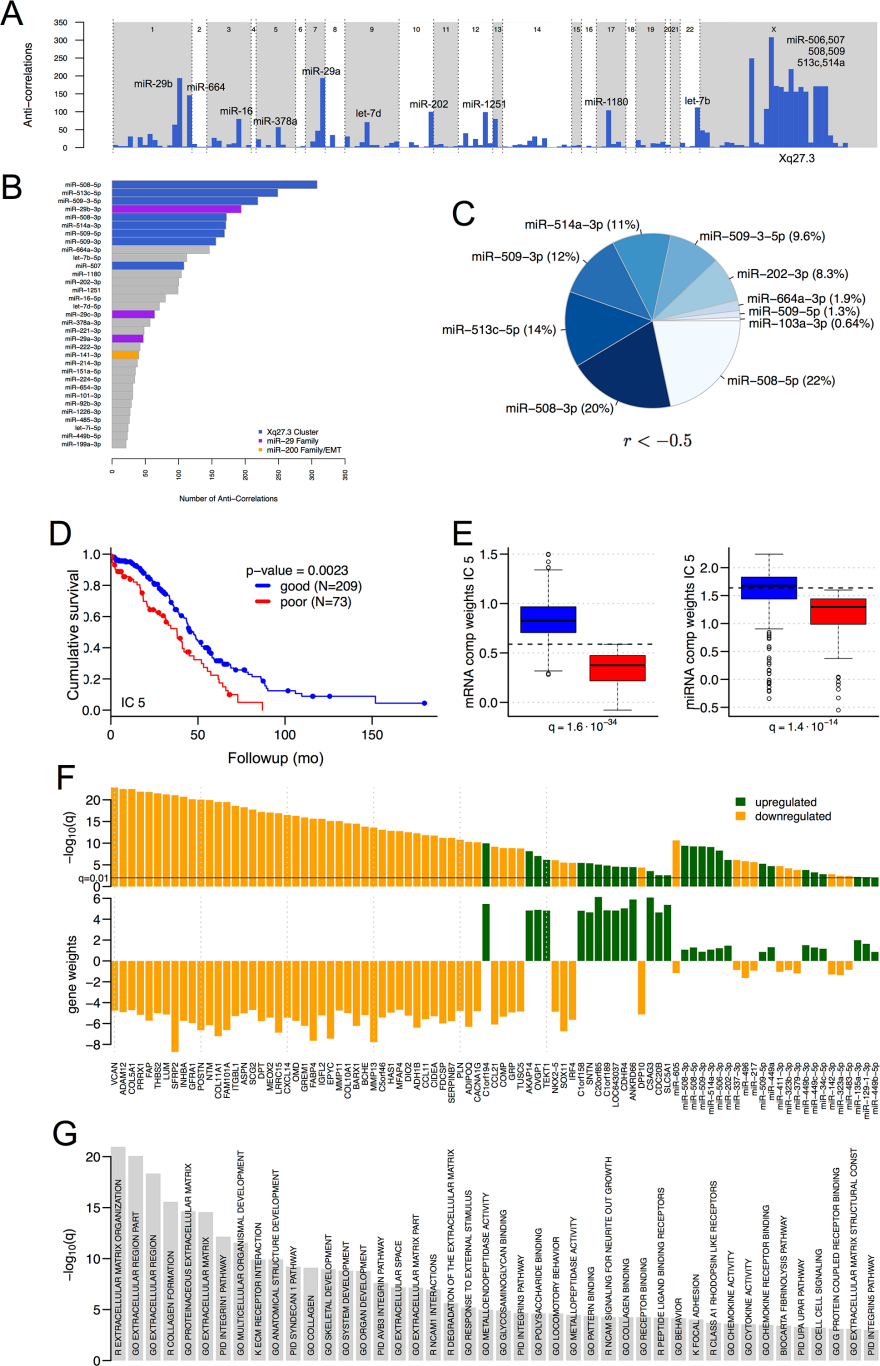
Significant miRNAs, mRNAs and pathways (**A**) Schematic transcriptome-wide view of the number of significant (FDR < 0.05) anti-correlations per miRNA (Spearman *r* < −0.3, for miRNAs with at least one significant anti-correlation). miRNA locations are not to scale. (**B**) The miRNAs in (A), ordered by the number of miR-gene interactions, and thresholded at 20 interactions. (**C**) miR-gene interactions with Spearman correlation coefficients less than −0.5. (**D–G**) Independent component analysis. (D) Kaplan-Meier plot for overall survival when samples are segregated by high vs. low component weights in IC 5 (see text). (E) Component weights of IC 5, for samples with favorable vs. unfavorable survival, for the mixing matrices for mRNAs (left) and miRNAs (right). Because samples with favorable survival have larger component weights than samples with favorable vs. unfavorable survival in IC 5, mRNAs with negative gene weights in this IC will be downregulated in samples with favorable survival. (F) Gene weights and *q*-values for the mRNAs and miRNAs that have significant gene weights in IC 5 (thresholds α = 6 for mRNAs, 1 for miRNAs) and are differentially expressed between samples with favorable and unfavorable overall survival. Green vs. orange: upregulated vs. downregulated in samples with favorable survival. (G) Enriched GO terms and pathways for IC 5 genes (*q* < 0.001, hypergeometric test, FDR-correction). R and K indicate REACTOME and KEGG, respectively.

We identified mRNA transcript isoforms from the mRNA-seq data using reference-based assembly [10], and calculated TargetScan 6.0 [11] miRNA binding sites on the full length reconstructed transcripts. For the 293 tumor samples with both mRNA-seq and miRNA-seq data (Table S2), we identified anti-correlated miRNA:mRNA pairs by calculating Spearman correlation coefficients (r) between the abundance profiles for mRNA isoforms and for 5p and 3p miRNA strands, thresholding at a *q*-value (FDR) < 0.05 (Table S2). Consistent with previous work [12], the miR-9 family and miR-29a/b were important for interactions with correlations below −0.3. Stronger interactions were also enriched in members of a ~100-kb cluster in Xq27.3 that contains hsa-mir-506, 507, 508, 509, 510, 513 and 514 (Figures 1A–1C, and S1A), particularly for miR-506, miR-508, miR-509, miR-513 and miR-514. For the 250 target genes with the strongest anti-correlations to members of this cluster, enriched Gene Ontology terms highlighted the ECM, and enriched KEGG pathways included ECM-receptor interactions (FDR = 6E–8) and focal adhesion (FDR = 0.02) (Table S3).

We extended these results by using independent component analysis (ICA) [13] to retrieve significant associations between miRNAs, mRNAs, and pathways. ICA is robust to co-expressed genes that are not always co-regulated [14], and to anti-correlations between miRNAs and target mRNAs that are not always significant [15]. Independent components (ICs) have been shown to capture the differential regulation of biological processes and metabolic pathways in breast cancer [16], acute myeloid leukemia [17], and Alzheimer's disease [18].

Briefly, ICA linearly decomposes an expression matrix (E) into ICs by solving the matrix equation E = CM. The columns of the component matrix C are the ICs in which each gene has a weight, and the weight of each IC in the samples is contained in the rows of the mixing matrix M. For miRNA-mRNA interactions, the gene weights of targeted mRNAs should be inversely correlated with the gene weights of targeting miRNAs in an IC that captures regulation by miRNAs. To identify such anti-correlations we modified the R function fastICA, which is widely used for expression data [19]. The resulting method, miICA, considers mRNA and miRNA data simultaneously.

ICA returned five ICs (Table S4). Using each IC's component weights, i.e. the rows of *M_m_* and *M_mi_*, we divided the samples into groups with favorable vs. unfavorable survival or recurrence by identifying an optimal component weight threshold for a two-group Kaplan-Meier test (K-M test) against clinical outcomes [20]. IC 5 best discriminated samples by survival (*P* = 2.3E-3, Figures 1D, S1B and S1C). IC 5′s component weights were higher in samples with favorable survival (Figure 1E, K-W test, FDR correction), which means that mRNAs or miRNAs with positive gene weights in this component were more abundant in the group of samples with favorable survival (Figures 1C and S1D). IC 5 contained 65 mRNAs with very significant gene weights (Figure 1F, α = 6), all of which were also differentially expressed between samples with favorable and unfavorable survival (*q* < 0.05, K-W test, FDR correction). Since miRNAs generally had lower gene weights than mRNAs in IC 5, we used a lower threshold α cut-off (α = 1) to identify 62 miRNAs with significant gene weights in IC 5, 24 of which were also differentially expressed between samples with favorable and unfavorable survival (Figures 1F and S1D, *q* < 1.0E-2, K-W test, FDR correction). miRNAs from the Xq27.3 miR cluster were more abundant in samples with favorable survival and had positive gene weights in IC 5; thus, targets of these miRs that are relevant to HGSOC should be sought among mRNAs with negative gene weights in this IC.

Consistent with the correlation analysis, many of the most significantly enriched pathways for IC 5 (Figure 1G) involve genes related to the ECM and cell adhesion (Table S4, threshold α = 3, *q* < 1.0E-3, K-W test). In all of these pathways the majority of genes had negative gene weights that were anti-correlated with the IC 5 gene weights of members of the Xq27.3 miR cluster. Predicted targets of the cluster miRs were significantly overrepresented among genes with negative gene weights in IC 5 (Table S4), consistent with targets of the miR cluster being associated with the enriched pathways. Overall, these results indicate that members of the Xq27.3 miR cluster, and the significant genes from IC 5 that are annotated as members of significant pathways, may be associated with survival in HGSOC.

### The Xq27.3 miRNA cluster is predicted to target structural and regulatory ECM components, and EMT regulators

To characterize in more detail ECM components that may be targeted by miR cluster members, we compared 1368 predicted target genes, and the 541 significant IC 5 genes, to six functional groups that have been defined for core matrisome and ECM-associated genes [21] (Table S5). For the core matrisome, predicted targets included 38 of 200 glycoproteins (*P* = 5.3E-10), 17 of 45 collagens (*P* = 6.2E-10) and 14 of 36 proteoglycans (*P* = 1.3E-8). For ECM-affiliated genes, predicted targets included 23 of 177 affiliated proteins (*P* = 6.7E-4), 51 of 254 regulators (*P* = 8.0E-14), and 50 of 353 secreted factors (*P* = 4.8E-8). Similarly, IC 5 genes were enriched in all ECM functional gene groups (*P* = 6.6E-24 to 6.6E-8) except ECM-affiliated genes.

We then assessed ECM-rich gene sets [22] that were prognostic for overall survival or recurrence in microarray-based gene expression data from the original TCGA study and two independent studies [7, 23, 24]. We compared six sets of highest-ranked 100 genes from: a) networks based on co-expression, functional linkage, and L_2_ penalty, and b) survival and recurrence outcomes, to the functional ECM groups, predicted miR cluster targets, and IC 5 genes (Table S5). The six gene sets were typically enriched (*P* < 0.001) for ECM glycoproteins, collagens, regulators and secreted factors. The 1368 predicted miR cluster targets were enriched in the gene sets for overall survival (*P* = 4.2E-52 to 1.3E-22) and for recurrence (*P* = 1.2E-33 to 1.4E-12). Finally, the 541 IC 5 genes were enriched in all six prognostic gene sets (*P* = 1E-25 to 3E-80).

Our sequencing data also identified epithelial-mesenchymal transition (EMT) regulators [25] as predicted targets of members of the miR cluster and of the miR-200 family: *SNAI1* (miR-510-5p), *SNAI2* (miR-507, miR-200b-3p), *TWIST1* (miR-507, 508-3p, 509-3p), *TWIST2* (miR-141-3p, 29b-3p, 29c-3p), *ZEB1* (miR-141-3p, 200b-3p) and *ZEB2* (miR-200b-3p, 507, 508-5p and 3p, 513c-5p, 514b-5p). Significant IC 5 genes included *SNAI2*, *TWIST1*, *TWIST2* and *ZEB1* (Table S2).

Given these results, we assessed how miR-509-3p mimics influenced levels of transcripts associated with ECM and EMT in *TP53*-wild type HEYA8 and *TP53*-mutated OVCAR8 ovarian cancer cell lines, in which we carried out time-series qPCRs on selected genes. TaqMan assays confirmed that miR-509-3p increased ~10,000 fold in abundance at 72, 96 and 120 h following transient transfection of miR-509-3p mimics (Figure S3A). Of the ECM and cytoskeletal genes, *ACTA2*, *COL1A1* and *COL3A1* were downregulated in response to miR-509-3p mimics in both cell lines at 72, 96 and 120 h after transfection, which correspond to 0, 24 and 48 h time points of the migration/invasion experiments (Figure S3B to S3D). Treatment with miR-509-3p mimics resulted in *COL5A1* downregulation in HEYA8 but upregulation in OVCAR8, while *COL5A2* was not significantly affected by the mimic in HEYA8, and was not detected in OVCAR8. ECM genes *SPARC*, *FN1* and *PRRX1* were upregulated in response to miR-509-3p mimics in both cell lines. miR-509-3p mimics downregulated *TWIST* at all three time points in both cell lines, and more significantly in HEYA8 than in OVCAR8 at each time point. *SNAI2* was upregulated at all three time points in both cell lines, with fold changes generally more significant in OVCAR8 than in HEYA8.

### miR-509-3p is positively associated with clinical outcomes and is localized to tumor cells

**Figure 2:**
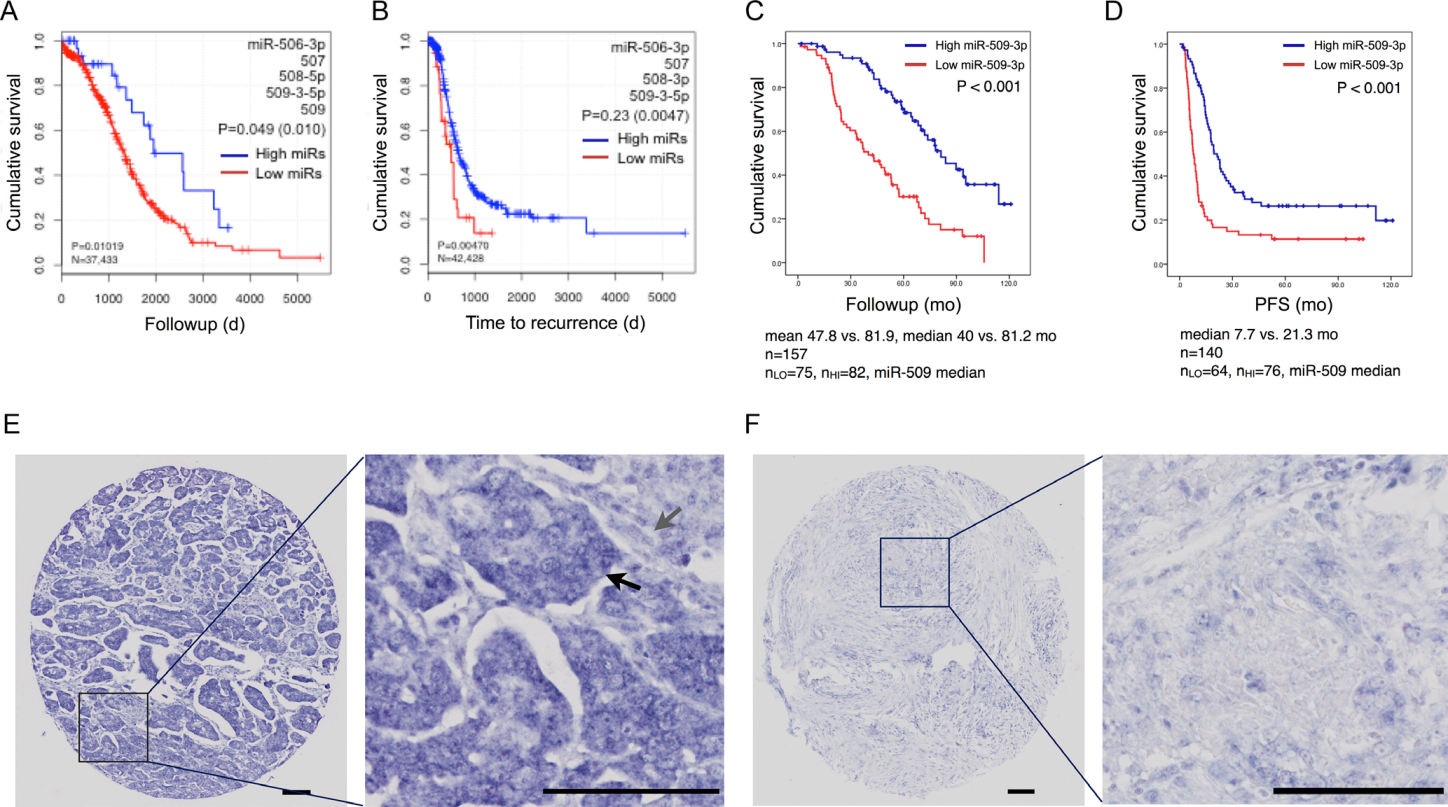
miR-509-3p and clinical outcomes (**A**, **B**) Kaplan-Meier plots for overall survival and time to recurrence for a discriminatory subset of Xq27.3 miRs, from TCGA sequencing data. (**C**, **D**) For an independent set of HGSOC samples, Kaplan-Meier plots for overall survival (*N* = 157) and progression-free time (*N* = 140) for sample groups separated by the median miR-509-3p *in situ* hybridization (ISH) score. (**E**, **F**) Representative ISH micrographs for miR-509-3p, showing (E) high (291) and (F) low (0) ISH staining intensities. Black and grey arrows indicate tumor and stroma cells respectively. Scale bars are 100 um.

For members of the Xq27.3 miR cluster, and the miR-29 and miR-200-families, we identified compact subsets of miRNA 5p and 3p strands whose abundance discriminated overall survival or time to recurrence. After correcting for multiple testing, only miRs from the Xq27.3 cluster remained statistically significant. For example, miR-506, 507, 508 and 509 collectively stratified a group of 37 samples by overall survival (*P* < 0.049) (Figures 2A, 2B and S2; Table S6).

From computational results, and the migration/invasion results described below, we focused on miR-509-3p. To confirm miR-509-3p's association with overall survival, and to determine whether the miR was expressed by cells within the tumor and/or the stromal cells from the tumor microenvironment, we used *in situ* hybridization to stain tissue microarrays for miR-509-3p in an independent set of 157 HGSOC samples (Table 1). We scored staining intensity in high-powered image fields in a blinded fashion by quantitative image analysis with CellProfiler v2.0 [26]. Consistent with results from sequencing data, overall survival was favorable (*P* < 1.0E-3) for samples with higher miR-509-3p expression levels (Figure 2C); progression-free survival was also favorable (*P* < 1.0E-3, Figure 2D). When miR-509-3p was detected, we found that it was largely localized within the tumor cells and not in the stroma (Figure 2E and 2F).

**Table 1:** Summary of independent HGSOC cohort used for ISH

Variable		miR-509-3p		*P*-value
		Low (*N* = 75)	High (*N* = 82)	
Tumour stage	I or II	4	9	0.21
	III or IV	70	73	
Cytoreduction	Optimal	41	56	0.11
	Suboptimal	30	24	
Recurrence or progression	Yes	57	57	3E-2
	No	8	21	
Median PFS (yr)		7.7	21.3	< 1.0E-3
Median OS (yr)		40	81.2	< 1.0E-3

For miR-509-3p, low and high refer to ISH staining intensities that are below and above the median intensity.

However, the ovarian cancer cell lines we tested had relatively low levels of miR-509-3p expression by qPCR (Figure S4A). The microarray-based miRNA abundance data in the NCI-60 cell lines (GEO: GSE26375) [27] includes seven ovarian cell lines (IGROV1, NCI/ADR-RES, OVCAR-3, OVCAR-4, OVCAR-5, OVCAR-8, SK-OV-3), five of which are included in the work that we report here. In these data too, the expression of miR-509-3p is low compared to other microRNAs such as miR-29abc and members of the miR-200 family (Figure S4B and S4C).

### miR-508-3p and miR-509-3p attenuate ovarian cancer cell migration and invasion

Because miR-508 and miR-509 were correlated with overall survival, and had predicted targets that were enriched for ECM components and for EMT regulators, we hypothesized that these miRNAs may be able to influence clinical outcomes in HGSOC by attenuating migration and invasion, and so metastatic progression. We assessed this *in vitro*, using a 96-well scratch-wound assay to compare HEYA8 and OVCAR8 cells that were untreated, versus those treated with either miRNA mimics from the Xq27.3 miR cluster or with a scrambled negative control (SCR) sequence.

**Figure 3:**
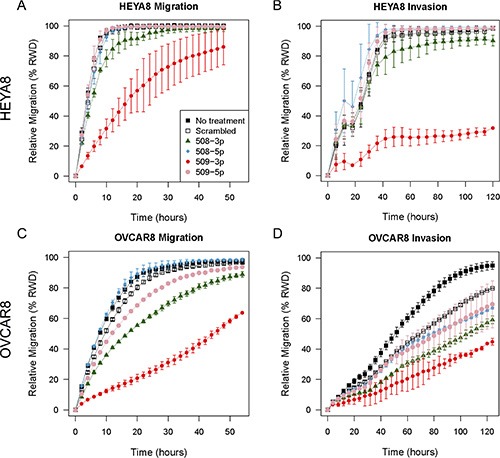
*In vitro* 96-well migration and invasion results after transfecting miR mimics into HEYA8 and OVCAR8 cells (**A**) Migration and (**B**) invasion for HEYA8 cells (*N* = 3). (**C**) Migration and (**D**) invasion for OVCAR8 cells (*N* = 3). Error bars show standard deviations. The time courses start 72 h after transfection.

Transient transfection with miR-509-3p strongly attenuated the ability of cells to migrate (Figure 3A and 3C) and invade a Matrigel matrix in both cell lines (Figure 3B and 3D). The relative wound density (RWD) of migration at 24 h was 39% lower in HEYA8 (p53-wild type) (*P* = 5.3E-13) and 64% lower in OVCAR8 (p53-mutant) cells (*P* = 5.8E-13) than in the same cells treated with the scrambled negative control sequence. Transient transfection with miR-508-3p had a smaller effect on migration for HEYA8 cells (6.3% reduction in RWD at 24 h, *P* = 0.20), and an intermediate effect in OVCAR8 cells (25% reduction in RWD at 24 h, *P* = 5.8E-13). In contrast, miR-509-5p only slightly reduced migration, as shown by the 11% decrease in RWD at 24 h for OVCAR8 cells (*P* = 6.6E-4), and showed no significant effect in HEYA8 (*P* = 1.00). The ability of cells to invade a Matrigel matrix was also most strongly attenuated by miR-509-3p, with a 70% reduction in RWD for HEYA8 (*P* = 5.4E-13), and a 29% reduction in OVCAR8 compared to SCR (*P* = 3.0E-3). miR-508-3p also reduced invasion, as indicated by the 6% decrease in RWD for HEYA8 (*P* = 1.9E-3) and 18% in OVCAR8 (*P* = 1.3E-2) and miR-508-5p did not significantly affect invasion for OVCAR8 (*P* = 0.89) or HEYA8 cells (*P* = 0.15). In contrast, miR-509-5p reduced invasion in OVCAR8 (14% reduction in RWD at 24 h, *P* = 3.2E-6), but did not significantly affect migration in HEYA8 cells (*P* = 1.0).

In independent experiments, we confirmed that miR-509-3p mimics attenuated migration strongly in OVCAR8 and SKOV3 and moderately in OVCAR3, OVCAR4, OVCAR5 and HEYA8 (Figures 5A, S5). Since the basal level of miR-509-3p in cells was very low in all six ovarian cancer cell lines tested (Figure S4A), the miR-509-3p inhibitor did not affect migration in these cell lines (Figure 6A).

**Figure 4:**
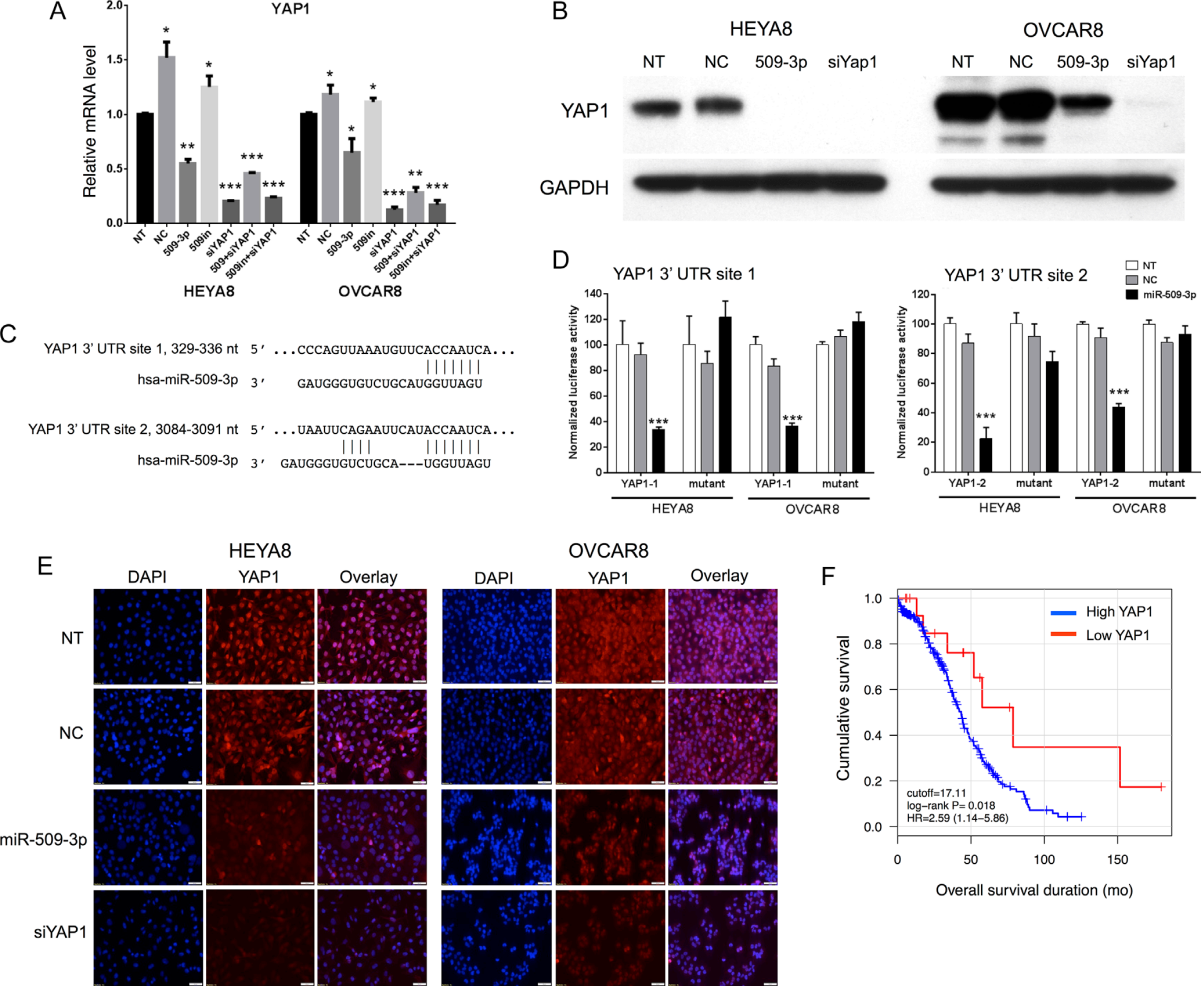
YAP1 target validation, and correlation with survival and migration (**A**) Relative mRNA transcript levels by qPCR and (**B**) protein levels by Western blot after miR-509-3p transfection and siRNA for *YAP1*, for HEYA8 (H8) and OVCAR8 (O8) cell lines. Error bars represent standard deviations. *, **, and *** indicate *P* < 0.1, 0.01 and 0.001. (**C**) Predicted binding sites in the *YAP1* 3′ UTR. (**D**) *YAP1* luciferase reporter activity for the two predicted binding sites, 48 h after transfecting a miR-509-3p mimic into HEYA8 and OVCAR8 cells. NT = no treatment. miR-NC = scrambled miR-509-3p mimic. Error bars represent standard deviations. *** indicates *P* < 0.001. (**E**) Yap1 immunofluorescence staining (middle panel) of non-transfected (NT) HEYA8 and OVCAR8 cells or cells transfected with miR-509-3p, *Yap1* siRNA (siYap1) or a negative control scrambled miRNA (NC) for 72 hr. Cell nuclei were stained with DAPI (left panel). Scale bars represent 50 um. (**F**) Kaplan-Meier curves for overall patient survival for high vs. low *YAP1* abundance, using TCGA sequencing data and a 17 RPKM threshold [20].

**Figure 5:**
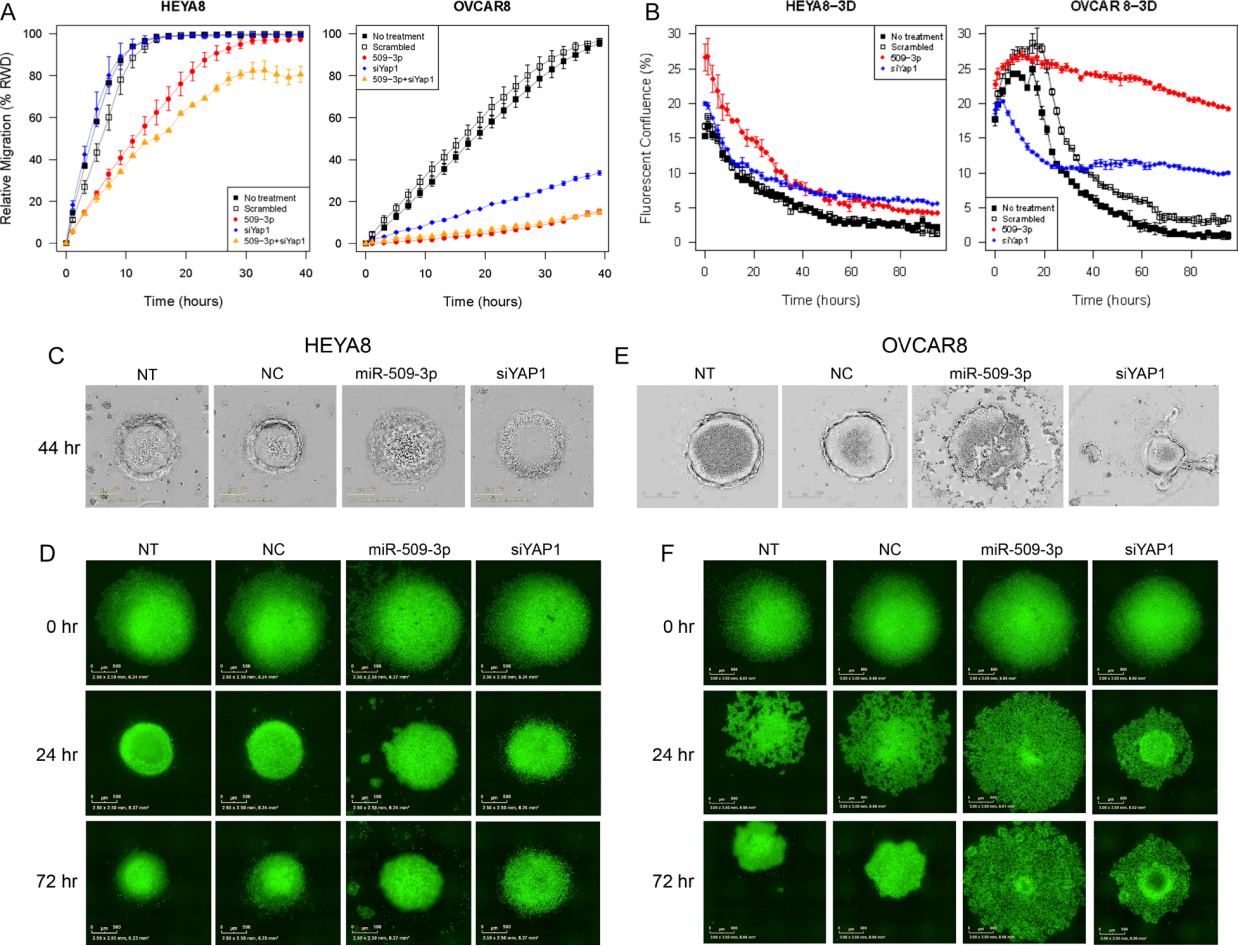
The effect of miR-509-3p and siYAP1 on cell migration and 3D aggregate formation for HEYA8 (left) and OVCAR8 (right) cells (**A**) *In vitro* 96-well migration time-course results for miR-509-3p transfection and for siRNA for *YAP1*, in HEYA8 and OVCAR8 cells. (**B**) Time course evolution of spheroidal aggregation. (**C**, **D**) Phase contrast and (**E**, **F**) fluorescence micrographs of time-course experiments. For treated cases in (A–F), time courses start (0 hr) 72 h after transfection. NT: no treatment. NC: treatment with a scrambled miR-509-3p mimic.

**Figure 6:**
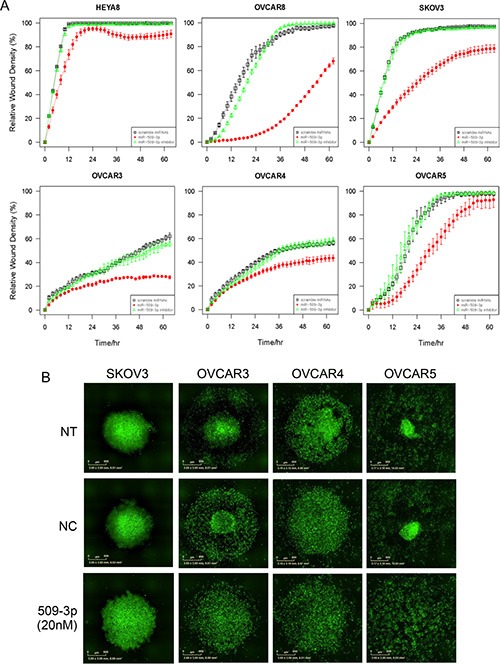
The effect of miR-509-3p and siYAP1 on migration and 3D aggregate formation for SKOV3, OVCAR3, 4 and 5 cell lines (**A**) *In vitro* 96-well migration time-course results for miR-509-3p transfection and siRNA for YAP1. The time courses start 72 h after transfection. (**B**) Fluorescence micrographs of time course evolution of spheroidal aggregation. NT: no treatment. NC: treatment with a scrambled miR-509-3p mimic.

### miR-509-3p influences transcript levels of genes involved in migration and invasion, and directly targets YAP1

We identified which of 143 miR-509-3p target genes predicted by TargetScan v6.2 (www.targetscan.org) have been reported as involved in cellular migration and invasion, then used quantitative RT-PCR with gene-specific primers to evaluate transcript levels for the predicted targets *BCAR1* [28], *GPC6* [29], *KCNMA1* [30], *PBX3* [31], and *YAP1* [32, 33]. Cells transfected with miR-509-3p mimics showed strong down-regulation of four of the five genes in OVCAR8 cells and three of the five genes in HEYA8 cells, relative to SCR treated and untreated controls (Figure S3E and S3F). Because *YAP1* is an oncogene in ovarian cancer, and a high *YAP1* level is associated with poor ovarian survival in TCGA data (Figure 4F), our independent cohort of 157 patients and in an independent study [34], we characterized *YAP1* in more detail.

*YAP1* is a transcriptional co-activator and key downstream nuclear effector of the Hippo signaling pathway [32, 33]. YAP1 protein level was downregulated in response to miR-509-3p mimics in both HEYA8 and OVCAR8 cell lines at 72, 96 and 120 h after transfection (Figure S3G). To demonstrate that the gene is a critical downstream mediator of miR-509-3p, we first showed that the downregulation seen from qPCR, Western blots and immunofluorescence staining (Figure 4A, 4B and 4E) is due to direct repression through one or both of the two predicted miR-509-3p binding sites in the *YAP1* 3′ UTR (Figure 4C). In luciferase reporter assays, both predicted target sites responded independently to miR-509-3p mimics (Figure 4D), and a mutation at either site was sufficient to abrogate miR-509-3p-mediated repression of the reporter. Then, we confirmed that low *YAP1* abundance was associated (*P* = 0.018) with better survival in the TCGA cohort (Figure 4F) [20]. Finally, we found that siRNA to *YAP1* impaired migration approximately 90% in OVCAR8 as effectively as miR-509-3p (Figure 5A). However, siYAP1 had no observable effect on migration in HEYA8 (Figure 5A), SKOV3, OVCAR3, OVCAR4 and OVCAR5 (Figure 6A). Our qPCR and Western blots showed that miR-509-3p repression on *YAP1* was largely at the protein rather than the transcript level (Figure 4A, 4B and Figure S6A, S6B). We also found that the level of YAP1 protein was over 2.4 fold higher in OVCAR8 than in HEYA8, SKOV3, OVCAR3, OVCAR4 and OVCAR5 (Figure S6C). Consistent with this, miR-509-3p had the largest influence on cellular migration in OVCAR8, and only marginal influence in the other cell lines, all of which expressed moderate to low levels of *YAP1*. We performed a rescue experiment to show that overexpression of exogenous *YAP1* largely reversed the inhibitory effects of miR-509-3p on migration in OVCAR8 cells. OVCAR8 cells co-transfected with a miR-509-3p mimic and a *YAP1* overexpression plasmid (pCDNA-FLAG-YAP1) had migrated 158% faster at 40 h than cells co-transfected with a miR-509-3p mimic and an empty vector C-FLAG-pCDNA3 (*P* = 0.040) (Figure S7). These data suggests that *YAP1* is likely an important prognostic marker for EOC and that miR-509-3p/*YAP1* axis may provide new druggable targets for ovarian tumors with high levels of *YAP1*.

### miR-509-3p mimics and YAP1 siRNA impair spheroid formation in 3D culture models of ovarian cancer

In ovarian cancer, compact multicellular spheroids are important in metastasis, which occurs primarily through the peritoneal fluid or ascites [35]. We used a magnetic levitation-based 3D culturing system [36, 37] and image analysis of time-course phase contrast and fluorescence micrographs to evaluate the impact of miR-509-3p and *YAP1* on spheroid formation in HEYA8 and OVCAR8 cells (Figure 5B–5F). We compared untreated (NT) to cells treated with a negative control (NC), miR-509-3p or siRNA to *YAP1*, starting time courses 72 h after transfection. As we had seen for migration and invasion, the treatments had different effects on the two cell lines. For HEYA8 cells, miR-509-3p and siYAP1 treatments attenuated aggregation moderately more than NT and NC treatments (Figure 5B–5D). For OVCAR8 cells miR-509-3p treatment strongly attenuated cellular aggregation into spheroids, with treated cells remaining largely dispersed, while siYAP1 treatment attenuated aggregation at a level intermediate between NC and miR-509-3p treatment (Figure 5B, 5E and 5F). We confirmed that spheroid formation was also attenuated in SKOV3, OVCAR3, OVCAR4 and OVCAR5 cell lines, with the magnitude of the attenuation varying between cell lines (Figure 6B). We also tested miR-508-3p, miR-508-5p, miR-509-3p and miR-509-5p effects on spheroid formation (Figure S8); of these, only miR-509-3p attenuated cellular aggregation into spheroids in OVCAR8 cells. This is consistent with our results showing that, of the Xq27.3 cluster members, miR-509-3p was the most clinically significant in relation to overall survival of ovarian cancer patients.

## DISCUSSION

In the work reported here, we identified microRNA-509-3p as a clinically significant microRNA by integrating functional assays and clinical outcomes with microRNA and RNA sequencing data from over 250 tumors from The Cancer Genome Atlas (TCGA) ovarian cancer cohort. We used *in situ* hybridization (ISH) to confirm the association of miR-509-3p with survival in an independent cohort of 157 ovarian cancer tumors. miR-509-3p attenuated cellular migration and spheroid formation in all six ovarian cancer cell lines tested. In ovarian cancer cell lines, our results established that miR-509-3p can functionally alter *YAP1* levels, and can attenuate migration, invasion, and aggregation into 3D spheroids.

miR-509-3p is expressed from a ~100-kb genomic cluster of miRNAs on Xq27.3. Members of this miR cluster have been associated with stage and survival in ovarian cancer, and are less abundant in omental metastases than in primary tumors [38–46]. In renal cancer, cluster members are less abundant than in adjacent normal tissue, and overexpression can reduce proliferation and migration, while inducing apoptosis [47, 48]. In contrast, in melanoma, low abundance of cluster members is associated with impaired growth, invasiveness and colony formation, and with increased apoptosis [49]. Recently, miR-509-3p was confirmed to directly target *XIAP* and inhibit proliferation and increase sensitivity to cisplatin in chemoresistant ovarian cancer cells [50]. Members of the Xq27.3 miR cluster have been reported to be strongly anti-correlated to a multi-cancer ‘metastasis-associated fibroblast’ gene signature [51]. miR-509-3p has recently been shown to target *CDK2* and to influence the cell cycle, colony formation and migration in human epithelial lung and cervical cancer cell lines [52]. Another family member, miR-506, and miR-200-family members, have been associated with EMT in a mesenchymal ovarian cancer subtype using the published microarray-based data [7, 43]. miR-509-3p and other Xq27.3 cluster miRs have recently been reported [53] as more abundant in a high-grade serous subtype (C5) that is defined by genes expressed in mesenchymal development [24], and less abundant in a subtype (C1) that has the poorest overall survival and is enriched in stromal genes.

Our results show that *YAP1* is a direct downstream target of miR-509-3p, and is both a major effector of miR-509-3p-mediated attenuation of migration and invasion, and an important effector in spheroid formation in ovarian cancer cells that contain higher levels of *YAP1*. *YAP1*, on 11q22, is frequently subject to copy number gains, mutations, and inappropriate expression in diverse types of cancer, and is considered an oncogene in ovarian and other cancers [34, 54–60]. Activation of *YAP1* in ovarian tumors is negatively correlated with clinical outcomes and response to taxanes [61].

Curiously, while *YAP1* is associated with the Hippo pathway, the mRNAs that were strongly inversely correlated to miR-509-3p in TCGA ovarian tumors were enriched not for Hippo pathway genes but for structural and regulatory components of the ECM. *YAP1* is functionally associated with the ECM through its non-canonical, Hippo-independent role as a mechanotransducer, and, as a central effector in mechanotransduction, *YAP1* influences how tumor cells sense and respond to the mechanical properties of the ECM and to their microenvironment [32, 33]. ECM changes have been reported to influence cell proliferation, differentiation, migration and invasion during normal development and differentiation [62], and mutations in mechanotransduction mediators, which are sensed by the cell as perturbations in ECM stiffness, have been associated with a number of diseases, including cancer [32, 33]. ECM rigidity and *YAP1* activation can interact to maintain cancer-associated fibroblasts [63].

A tightly regulated balance between ECM deposition and turnover is critical for tissue homeostasis, and can be impaired in cancer cells, influencing migration, invasion and metastatic progression [35]. This is especially relevant to epithelial ovarian cancer, which primarily metastasizes by cells and multicellular spheroids exfoliating into the peritoneal fluid [35]. Multi-cellular spheroids can be drug resistant, due in part to the decreased penetrance of therapeutics to the protected interior cells [64]. Spheroid formation allows cancer cells to overcome anoikis, a form of apoptosis that can be triggered in individual cells that are unable to attach to other cells or to the ECM [65].

**Figure 7:**
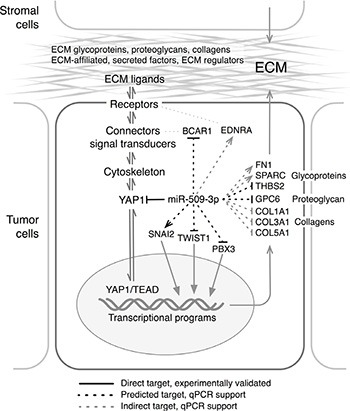
Model for the proposed mechanisms through which miR-509-3p alters the YAP1/ECM axis Arrows and Tees indicate genes that were respectively up- and down-regulated in response to miR-509-3p (Figure S2). ECM functional gene groups are from [21], and names along the ECM/*YAP1* axis were adapted from Figure 1 in [75].

The model in Figure 7 summarizes our findings for miR-509-3p and the *YAP1*/ECM axis. Collectively, our results suggest that miR-509-3p-mediated changes in levels of *YAP1* and ECM genes impair migration, invasion, and spheroid formation and so may attenuate metastatic progression in advanced stage ovarian cancer. Further, our results suggest that the direct downstream miR-509-3p target *YAP1* is likely a critical driver of cellular migration and spheroid formation in ovarian cancers in which levels of YAP1 protein are high. We suggest that miR-509-3p and drugs that target the miR-509-3p/*YAP1*/ECM axis may offer novel therapeutic opportunities in ovarian cancer, and potentially in other cancers.

## MATERIALS AND METHODS

### Library construction, sequencing, data processing

mRNA and miRNA sequencing data were generated using methods previously described [66]. For miRNA abundance we used a 5p and 3p mature strand data representation.

### Analysis data sets

Analyses involving mRNA-seq and miRNA-seq used 289 datasets present in both data types. For miRNA-seq, unsupervised clustering, subset analysis and differential abundance calculations used data for 475 tumor samples. The hg19 (GRCh37) reference human genome was used for both data types. See Table S1.

### miR-to-gene correlations

As described recently [67], we calculated correlations for RPM data for each miRNA-seq 5p or 3p strand and each mRNA-seq transcript from reference-based assembly [10]. The *P*-values obtained from the Spearman correlations were corrected for multiple testing using the Benjamini Hochberg method. Corrections for multiple testing were done per miRNA, and a *q*-value < 0.05 significance threshold was applied. TargetScan v6.0 binding site predictions [11] were calculated on the full-length sequences of the predicted transcripts. Enriched GO process, function and cellular locations, and enriched KEGG pathways, were calculated using DAVID [68] and mgsa [69].

### Independent component analysis, miICA

In RPKM (mRNAs) and RPM (miRNA 5p or 3p strand) expression matrices, genes and miRs with a maximum RPKM or RPM < 10 respectively were removed, and only samples with survival data and both mRNA and miRNA expression profiles were retained in the expression matrices (denoted *E_m_* and *E_mi_* respectively). For genes for which the expression matrix generated from reference-based assembly contains results for more than one splice variant, we sum the expression for the individual spice-variants across the samples, generating a matrix of total mRNA expression that contains 12664 mRNAs across the 283 samples. We use the R-function rm.outlier() to remove outlier mRNAs and miRNAs with a coefficient of variation g (i.e. standard deviation divided by mean expression) greater than 5. As is done when ICA is applied to expression matrices from microarray experiments, we log2-transformed the miRNA and mRNA expression matrices, substituting small values for zeros.

Independent component analysis (ICA) [13] decomposes an expression matrix (*E*, GxS) into a component matrix (*C*) and a mixing matrix (*M*), i.e.

$$E=CM\text{ or }E_{gs}=\overset{1}{\underset{i=i}{\sum }}C_{gi}M_{is}$$

where *C* is the GxI component matrix containing the independent components (ICs) in the columns, and *M* is the IxS mixing matrix giving the linear mixing of each IC in each sample. The convention is to scale the ICs so that in each IC the entries *C_gi_* have a mean of zero and a variance of one.

Component weights *M_is_* measure the activity level of transcriptional programs, e.g. biological processes, in an IC across the samples. For example, when an IC maps to a cancer-related pathway, then this IC's weights will be different in cancerous samples vs. non-cancerous samples. Component weights can be used to classify samples according to physiological, histological, or other features [70, 71]. Regarding gene weights *C_gi_*, if one gene is inhibited by a transcriptional program while another is activated, then their gene weights will be anti-correlated, e.g. one being negative and the other positive.

Before applying ICA, the number of independent components (ICs), *I* ≤ *min (N, S)*, needs to be specified. To determine the optimal number of ICs to be estimated we used random matrix theory, as implemented in the EstDimRMT() R-function from the isva (v1.3) R package. The maximum of EstDimRMT() applied to the miRNA and the mRNA expression matrices, separately, was used as the number of ICs (*I*) to be estimated.

We used the default fastICA algorithm (v1.1–16) to first pre-whiten the expression matrix (*E*) by projecting it onto the first *I* principal components in the matrix (*K*). Subsequently we estimated an un-mixing matrix *W* such that *C = EKW* contains columns that are maximally independent, i.e. the independent components.

We extended the fastICA package to address genes and miRNAs simultaneously, i.e. to enable estimating an un-mixing matrix that simultaneously fulfills

$$\begin{array}{l} C_{m}\;=\;E_{m}K_{m}W\text{ and }C_{mi}\;=E_{mi}K_{mi}W \end{array}$$

and maximizes the independence of the ICs contained in both C_m_ and C_mi_. Subscripts *m* and *mi* denote mRNA and miRNA, respectively. The mixing matrices are calculated from the estimated un-mixing matrix by solving

$$\begin{array}{l} C_{m}\;M_{m}\;=\;E_{m}K_{m}W\;{\left(K_{m}W\right)}^{-1}\;and \end{array}$$

$$\begin{array}{l} C_{mi}\;M_{mi}\;=\;E_{mi}K_{mi}W\;{\left(K_{mi}W\right)}^{-1} \end{array}$$

for *M_m_* and *M_mi_*.

For the ICs to be independent they must have a maximum negentropy [13, 72], i.e. the distribution of the entries C_gi_ in each IC is non-Gaussian. fastICA, and our modified fastICA, iteratively maximize the negentropy of the ICs using random seeding. Depending on the initial random guess for the un-mixing matrix, the ICs that do not represent structures in the data may converge to local, rather than to global maxima of negentropy. However, ICA is expected to always extract an IC that identifies structures in the data, i.e. to always converge to approximately the same negentropy maximum. To quantify this we run the modified fastICA 30 times. For each run, we calculate Spearman's correlation coefficients between each of the original ICs and the ICs in the 29 other runs (algorithm adapted from [73]), and store the highest absolute correlation coefficient calculated for each IC in run r. We average the stored absolute correlation coefficients component-wise, take the average correlation coefficients as a measure for the stability of an IC, and order ICs according to their stability. We applied our modified fastICA() to the expression matrices containing the 283 samples that had survival and progression data, as well as miRNA and mRNA expression profiles

Following ICA, one way to identify significant genes in an IC is by finding genes that satisfy the inequality

$$\begin{array}{l} \left|G_{gi}\right|\geq \;\alpha \;\cdot \;SD\;\left|C_{i}\right| \end{array}$$

where *SD*|*C_.i_|* is the standard deviation of the absolute gene weights in IC *i*, and α is often set to a value between 2 and 3 [16]. Here we use α = 3. Because we are interested in the biological processes that are related to the survival and recurrence times, for each IC we determine an optimal cutoff for the component weights by using Cutoff Finder [20] v2.0 to divide the patients into good and poor survival or progression groups.

We performed a pathway analysis on each IC by testing for overrepresentation of pathway gene-sets from the MsigDB (v3.0) (broadinstitute.org/gsea/msigdb/). Denoting the total number of genes annotated for a specific pathway (*P*) by *G_p_*, and the number of significant genes in IC *i* that are annotated by *P* as *K_p,I_*, we apply a hypergeometric test to test whether the probability *P(X > K_p,i_)* is significant when there are *N* genes in total and *N_i_* significant genes in IC *i*.

All *p*-values are FDR-corrected using the R function p.adjust.

For miR target enrichment calculations, a miR binding site was taken to be a 7mer seed match in the longest annotated Ensembl vGRCh37.p11 3′UTR. Statistical significance was calculated using *q*-values for a hypergeometric test.

### miR subsets that discriminate clinical outcomes

To identify a concise set of miRNA mature strands whose expression was strongly associated with overall survival, we extracted combinatorial subsets of all mature strands from members of the Xq27.3 miR cluster, and from the miR-29 and miR-200-families, separately, and tested each subset for its log-rank *P*-value using the R survival package. For each of the three sets of 5p and 3p strands, we generated all possible subsets, and for each subset we separated samples into two groups using k-means clustering on abundance (RPM) data that we normalized between samples, and then again between miRNAs, such that the range of abundance was not a factor in the clustering. We tested Kaplan-Meier (KM) curves for each pair of sample groups for statistical significance, correcting log-rank *P*-values for multiple testing using the Benjamini Hochberg method. Representative subsets for which we displayed KM plots had a both high-ranked significant KM log-rank *P*-value and relatively large numbers of samples in the smaller of the two sample groups.

### Cell cultures and transient transfections

Ovarian cancer cell lines OVCAR8 and HEYA8 were maintained in RPMI media (Life Technologies) supplemented with 10% heat inactivated fetal bovine serum (Atlanta Biologicals) and 1% of penicillin/streptomycin. Cell lines were incubated at 37°C with 90% humidity and 5% CO_2_. miRNA mimics/YAP1 siRNA (Life Technologies) and negative controls (Life Technologies) were transiently transfected at a 20 nM concentration using Lipofectamine 3000 (Life Technologies) following the manufacturer's recommendations. For rescue experiments, pCDNA Flag-Yap1/empty vector C-FLAG-pCDNA3 were transfected at 3.6 ug/well in a 6-well plate using FuGene HD (Promega) 24 hrs after miRNA transfection, following the manufacturer's recommendations.

### Scratch-wound migration assay

Scratch-wound assays were performed using the Essen BioScience Cell Player Migration System. Seventy-two h after transfection, cells were seeded in 96-well ImageLock microplates (Essen BioScience) at 35,000 cells per well. Artificial scratch wounds were generated using the 96-well WoundMaker™. Each well was washed twice with PBS, and then overlaid with 200 μL of RPMI 1640 medium supplemented with 10% FBS. Images were acquired using the IncuCyte-FLR™ live cell imaging platform. Cell migration was calculated with IncuCyte's automated image analysis algorithm using the Relative Wound Density (RWD) metric, which is defined by

$$\begin{array}{l} RWD\;\left(t\right)\;=\;100\frac{w\left(t\right)\;-\;w\;\left(0\right)}{c\left(t\right)\;-\;w\;\left(0\right)} \end{array}$$

where w(t) is the density of the wound region and c(t) is the density of the cell region at time t. Density was plotted as a function of time for three independent experiments, with error bars showing standard deviations. Statistical significance was determined using one-way ANOVA with the Tukey posthoc test (IBM SPSS Statistics 21). Results reported in Figures 3 and 5 were from independent experiments in two laboratories.

For the YAP1 overexpression rescue experiment, scratches were made using 200 ul pipette tips. Cells were washed two times by PBS and fresh media was added. The cell culture plates were transferred to a Biostation CT (Nikon) instrument programmed to take images every 4 h for up to 40 h. The images at different time points were aligned using the NIS-Element AR software. Scratch edges were detected and scratch area was masked with red color for all the images using NIS-Element AR software. Binary area fraction (fraction of masked area with red color) for each time point was measured using NIS-Element AR software. Area covered due to migration of cells after each time point was obtained by subtracting binary area fraction for each time point from that of initial time point. Average migration area for each time point was calculated for three biological replicates.

### Matrigel invasion assay

Cell invasion assays were performed using Matrigel-coated 96-well microplates. Scratch-wounds were generated as described for the scratch-wound migration assay, and 50 μL of Matrigel Matrix (BD Biosciences), diluted to 1 mg/mL in cell culture medium, was added to each well. Microplates were incubated at 37°C to set the Matrigel, and was overlaid with 200 μL of culture medium 24 h later. Density was plotted as a function of time for three independent experiments, with error bars showing standard error deviations.

### 3D culture assay

To form three dimensional (3D) cell aggregates, cells, with or without miRNA/siRNA transfection, were incubated overnight in RPMI with 10% FBS, with a magnetic gold–polymer–iron oxide hydrogel (Nanoshuttle-PL, NS) (n3D Biosciences, Houston TX) to allow the attachment of NS to the cells [36, 37, 74]. 5 uM of CellTracker Green CMFDA Dye (Molecular Probes) was added to the cells and incubated for 30 minutes. The cells were washed with PBS, trypsinized, resuspended in RPMI with 10% FBS, and then magnetically levitated to the air-water interface for 4 hours, where they formed 3D structures with an ECM. Next, the 3D structures were mechanically disrupted by pipetting, and the resulting suspensions of aggregates were transferred to a 96-well plate, where they were formed or ‘printed’ into either a dot or a ring shape by placing a dot or ring magnet ‘drive’ underneath the plate for 15 minutes. Using either phase contrast or fluorescence, the 3D culture area was automatically imaged over time by an IncuCyte ZOOM live-cell imaging system (Essen BioSciences, Ann Arbor, Michigan), and the area of the dense 3D spheroidal regions was recorded for each well.

### Quantitative polymerase chain reaction (qPCR)

Total RNA from cellular pellets was isolated using an miRNeasy Mini Kit (Qiagen) following the manufacturer's instructions. Purity and RNA concentration were measured using a ND-100 Nanodrop spectrophotometer (Thermo Scientific). Reverse transcription of mRNAs was performed using the TaqMan Reverse Transcription Reagents (Invitrogen) using 300 ng of total RNA for each 20 μL reaction. qPCR was performed using the SYBR Green reagent (Applied Biosystems) in a 10 μL reaction with 1 μL of cDNA and 0.005 nmol of primers. Reactions were run on a StepOnePlus Real-Time PCR system (Life Technologies) and analyzed using the delta-delta CT method. The level of 18S ribosomal RNA was used to normalize the relative expression of genes.

### *In situ* hybridization

A tissue microarray with high-grade serous ovarian cancer samples, as determined by a gynecologic pathologist, was used. Formalin-fixed paraffin-embedded tissue sections were dewaxed in xylene, and rehydrated through an ethanol dilution series. Tissue sections were digested with 15 μg/mL proteinase K for 20 min at room temperature, were then loaded onto a Discovery Ultra slide staining system (Ventana/Roche) for *in situ* hybridization. The tissue microarray slides were incubated with a double DIG-labeled miRCURY LNA microRNA probe (Exiqon) for 2 h. The digoxigenin was then detected with a polyclonal anti-DIG antibody and alkaline phosphatase-conjugated second antibody (Ventana) using NBT-BCIP as the substrate. We confirmed that ovarian tissue has very low background staining, using a double DIG-labeled LNA negative control probe (Exiqon) and the same procedure as above.

The expression levels for miR-509-3p were determined as previously described [26]. In brief, representative images from each tissue section were imaged at 10 × magnification. Blinded from all clinical data, CellProfiler 2.0 software [75] was trained with negative and positive controls to establish staining intensity threshold levels and cell diameter parameters for enumerating the number of positively stained cells at 100 × magnification. These parameters were used to score all images.

### Luciferase reporter assay

The DNA sequence, flanked by ~ 600 nucleotides, with the two predicted miR-509-3p target sites at -329 to -336 nt in the 3483-nt YAP1 3′UTR (YAP1_1: -329 bp to -336 bp, YAP1_2: -3084 bp to -3091bp) were PCR-amplified from human genomic DNA and sub-cloned into the NotI restriction site of a psiCHECK-2 vector (Promega) using the Gibson Assembly Cloning Kit (New England Biolabs). We generated mutant clones by mutating the miR-509-3p seed interaction sites in the reporter plasmid construct, using the Q5 Site-Directed Mutagenesis kit (New England Biolabs) for YAP1_1 (ACCAATCA->ACgAtTCA) and the QuickChange II XL Site-Directed Mutagenesis kit (Agilent Technologies) for YAP1_2 (ACCAATCA->gCCAAgCA). Both clones were verified by Sanger sequencing. 100ng of each reporter plasmid was transfected alone, or co-transfected with either 20 nM of a miR-509-3p mimic, or a scrambled negative control miRNA mimic, into HEYA8 and OVCAR8 cells, using lipofectamine 2000 (Life Technologies) at 10000 cells/well in a 96-well plate, following the manufacturer's recommended protocol. Luciferase assays were performed at 48 h post transfection using a Dual-Luciferase Reporter Assay System (Promega). Renilla luminescence signals were normalized by the firefly luciferase signals. Three replicates were performed for each assay.

### Relationship of YAP1 abundance and survival

An RPKM abundance threshold giving the most significant high-vs-low log-rank *P*-value for overall survival was identified with CutoffFinder v2.0 [20].

### ECM functional gene groups

Gene symbols for each ECM functional category [21] were taken from matrisome-table-Hs_CURRENT.xlsx, which was downloaded from web.mit.edu/hyneslab/matrisome.

### Statistical significance of overlap between two gene lists

*P*-values were calculated using a custom Mathematica (Wolfram Research, Champaign, IL) script that implemented the method described at nemates.org/MA/progs/representation.stats.html. For example, given a population of n_pop_ = 21929 genes, *d* = 1368 predicted target genes for the Xq27.3 cluster members and *n* = 200 ECM glycoproteins from matrisome-table-Hs_CURRENT.xlsx from web.mit.edu/hyneslab/matrisome [21] had an overlap of *x* = 38 genes, for which *P* = 6.3E-10.

### Immunofluorescence staining

Cells were seeded onto a Falcon culture slide (Corning) and cultured in complete RPMI medium under standard cell culture conditions. Cells were fixed in 4% paraformaldehyde at room temperature for 15 min, then were permeabilized in 1 × PBS containing 0.1% Tween-100 for 30 min at room temperature. The cells were blocked in a solution of 1 × PBS containing 10% normal goat serum and 0.1% Triton X-100 for 4 h. After being washed twice with 1 × PBS for 5 min each time, the cells were incubated overnight with a 1:200 dilution of monoclonal rabbit Yap1 antibody (Cell Signaling Technology) in the blocking solution at 4°C. After being washed twice with 1 × PBS for 5 min each time, a goat anti-rabbit IgG conjugated with Alexa Fluor 568 (Life Technologies) at 1:1000 in the blocking solution was incubated with the cells at room temperature for 1 hr. The cells were stained with DAPI for 5 min after being washed twice with 1 × PBS, and then mounted with ProLong Gold Antifade Mountant (Life Technologies). The fluorescence images were acquired using an Olympus imaging microscope with a 20 × objective.

### Western blot

Total proteins were extracted from cellular pellets using protein extraction buffer (Triton X-100 1%, NaCl 150 mM, Tris 25 mM, pH 7.6) with Complete mini protease inhibitors (Roche). Thirty micrograms of protein were run on Nupage-NOVEX Bis-Tris 4–12% gels using a MOPS buffer. After electrophoresis proteins were transferred to a PVDF membrane (Invitrogen), and the membranes were then blocked for 1 h at room temperature with 5% non-fat milk dissolved on TBS-T (50 mM Tris-HCl, 150 mM NaCl, 0.1% Tween 20, pH 7.5). Primary antibodies Yap1 and GAPDH (Cell Signaling Technology) were added at a concentration of 1:1000 and were incubated overnight with the membrane at 4°C. HRP-linked anti-rabbit secondary antibodies (Cell Signaling Technology) were added at a concentration of 1:10,000 and incubated with membrane at room temperature for 1 hr. The proteins were visualized using a Pierce ECL Plus chemiluminescent substrate (Thermo Scientific).

## SUPPLEMENTARY MATERIALS TABLES AND FIGURES














