# Correction: miR-509-3p is clinically significant and strongly attenuates cellular migration and multi-cellular spheroids in ovarian cancer

**DOI:** 10.18632/oncotarget.15921

**Published:** 2017-03-06

**Authors:** Yinghong Pan, Gordon Robertson, Lykke Pedersen, Emilia Lim, Anadulce Hernandez-Herrera, Amy C. Rowat, Sagar L. Patil, Clara K. Chan, Yunfei Wen, Xinna Zhang, Upal Basu-Roy, Alka Mansukhani, Andy Chu, Payal Sipahimalani, Reanne Bowlby, Denise Brooks, Nina Thiessen, Cristian Coarfa, Yussanne Ma, Richard A. Moore, Jacquie E. Schein, Andrew J. Mungall, Jinsong Liu, Chad V. Pecot, Anil K. Sood, Steven J.M. Jones, Marco A. Marra, Preethi H. Gunaratne

**Present:**

**ACKNOWLEDGMENTS**

The authors gratefully acknowledge the data production teams at the Genome Sciences Centre.

**Corrected:**

**ACKNOWLEDGMENTS**

The authors gratefully acknowledge the data production teams at the Genome Sciences Centre. The results published here are in whole or part based upon data generated by The Cancer Genome Atlas managed by the NCI and NHGRI. Information about TCGA can be found at http://cancergenome.nih.gov.

Original article: Oncotarget. 2016; 7(18):25930–48. doi: 10.18632/oncotarget.8412.

