# Supplementary material for: miR-509-3p is clinically significant and strongly attenuates cellular migration and multi-cellular spheroids in ovarian cancer
Source: Oncotarget. 2016 Mar 27;7(18):25930–48. doi: 10.18632/oncotarget.8412 (PMC5041955; doi:10.18632/oncotarget.8412)
Supplement: Supplementary file 1 [file oncotarget-07-25930-s001.pdf]

miR-509-3p is clinically significant and strongly attenuates cellular migration and multi-cellular spheroids in ovarian cancer

Supplementary Materials

Supplementary Table S1: TCGA identifier barcodes for samples. Samples include 300 mRNA-seq tumour samples, 475 miRNA-seq tumour samples, and the 293 samples for which both mRNA-seq and miRNA-seq data were available.

Supplementary Table S2: Spearman correlation coefficients and *q*-values for miR-mRNA correlations. Correlations were calculated on full-length expressed transcript sequences by a TargetScan 6.0 pipeline for mature 5p and 3p strands, for (a) miR-506-514a cluster members, (b) miR-200 family members, (c) miR29a,b,c, as well as (d) correlations with EMT regulators SNAI1/2, TWIST1/2 and ZEB1/2 for these miRNAs.

Supplementary Table S3: Enriched GO biological process terms for strongly anticorrelated predicted targets for miR-506-514a

Supplementary Table S4: Detailed results from independent component analysis with miICA

Supplementary Table S5: Detailed results from calculations for the significance of overlap between two gene sets

Supplementary Table S6: Detailed results from combinatorial Kaplan-Meier analysis. The analysis identified subsets of miRNAs that correlate strongly to overall survival or recurrence.

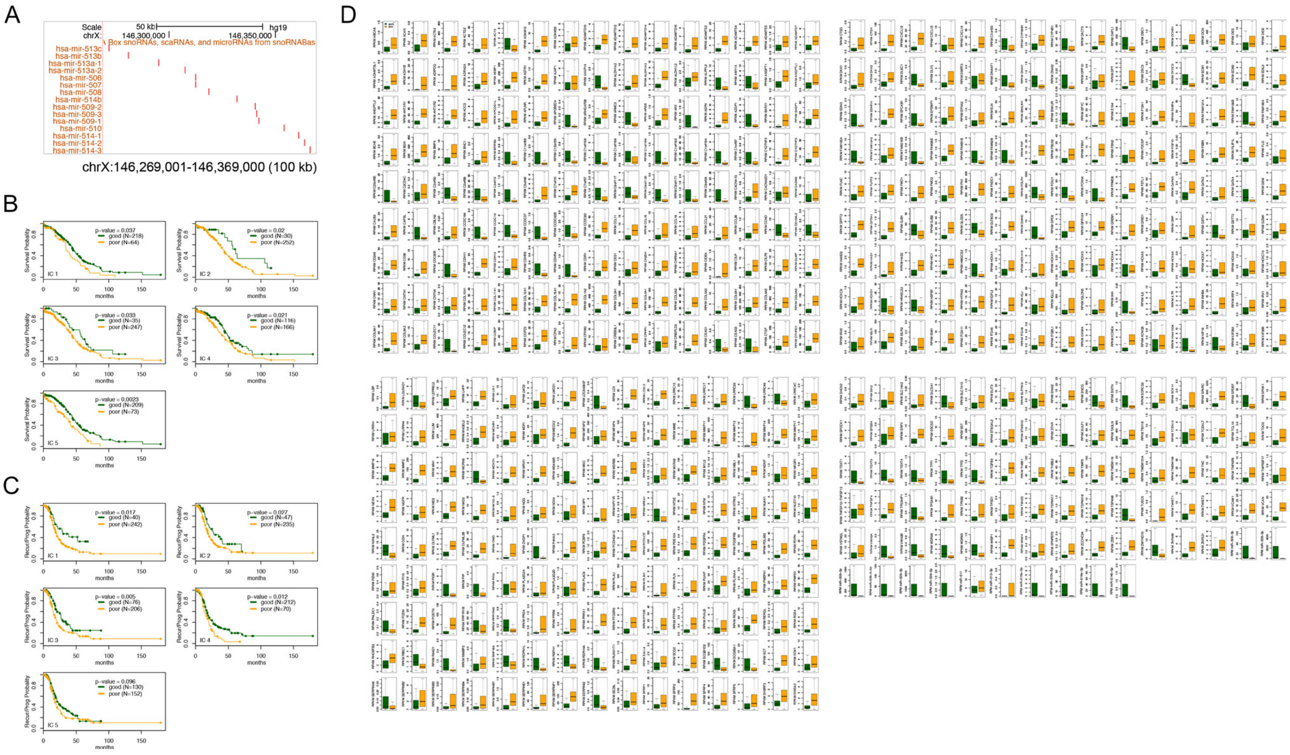

Supplementary Figure S1: Results from independent component analysis for the miR cluster in the TCGA cohort. (**A**) The miR-506-514 miR cluster on Xq27.3 (genome.ucsc.edu, hg19). (**B, C**) Kaplan-Meier plots for five independent components for (B) overall survival, (C) recurrence or progression. Gold and green respectively represent sample groups with unfavorable vs. favorable survival or recurrence. (**D**) Abundance distributions of significant IC 5 mRNAs and miRNA mature strands. These mRNAs and miRs had statistically significant gene weights and were differentially abundant between samples with favourable (green) and unfavourable (gold) survival.

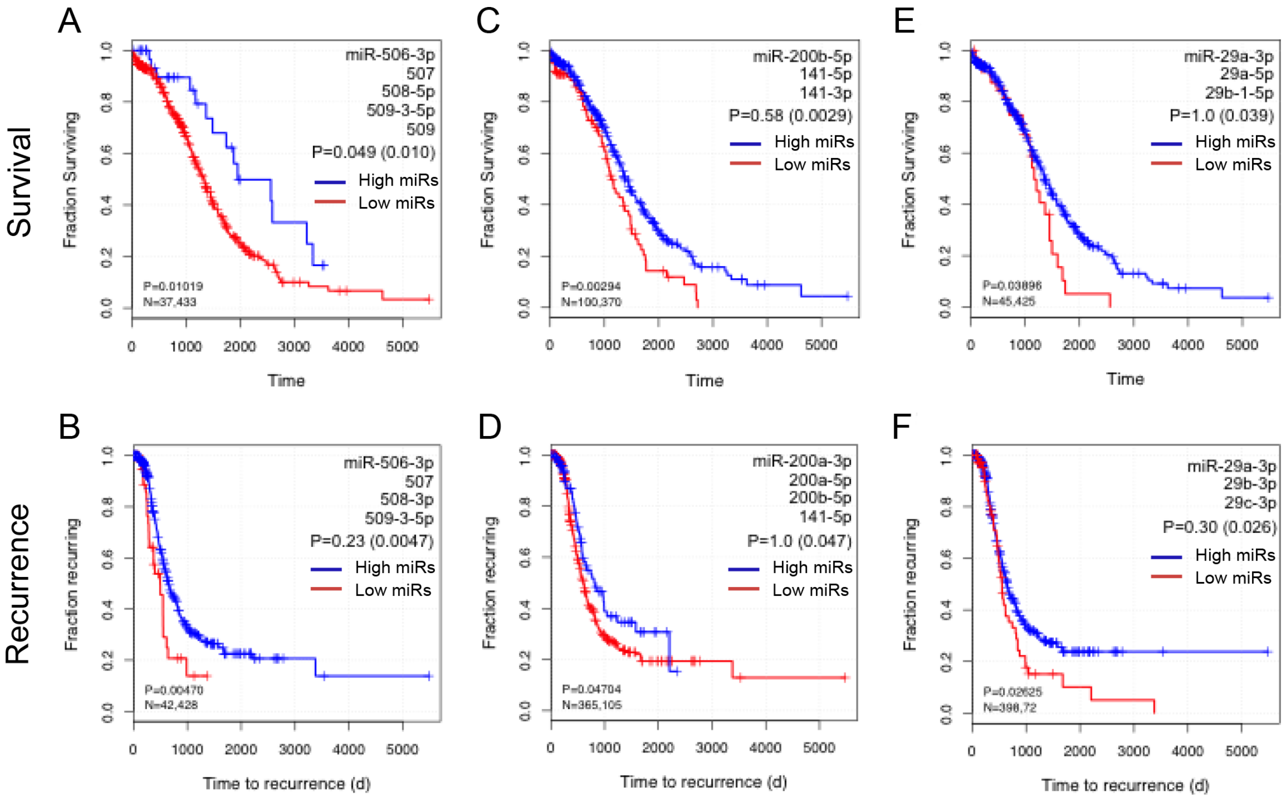

Supplementary Figure S2: Discriminatory subsets of miRNAs. (**A, B**) The Xq27.3 miR-cluster. (**C, D**) the mir-200 family. (**E, F**) mir-29abc. Above: overall survival, below: recurrence. Graphs show results for representative 5p/3p strand (i.e. miR) subsets from each combinatorial test (Methods). Text for each graph shows the miR subset, the log-rank *P*-value, and the number of samples in the two k-means groups (see Methods). Red vs. blue curves correspond to the k-means group with relatively high vs. low miR abundance, and to shorter vs. longer outcome times.

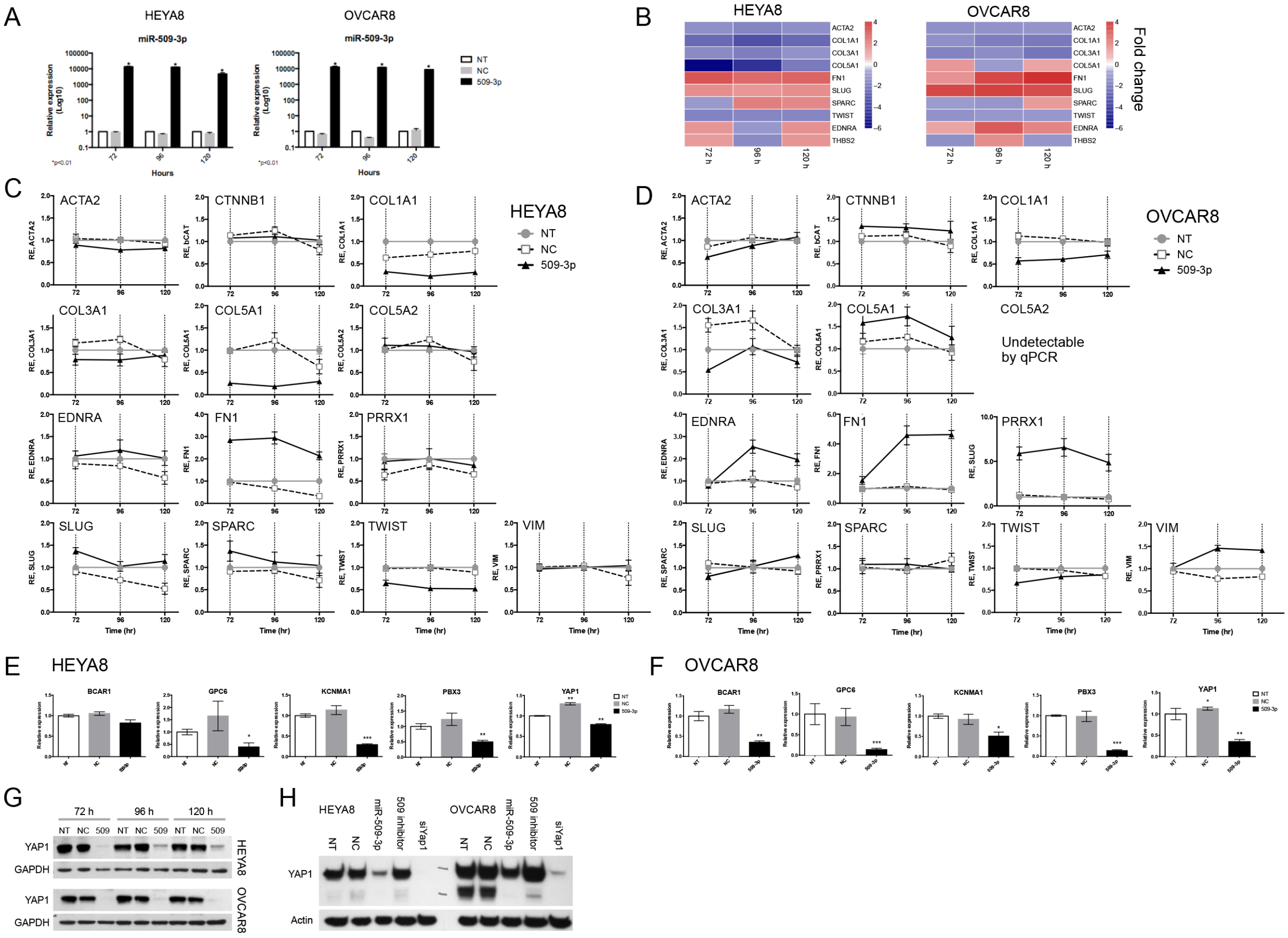

Supplementary Figure S3: Effects of miR-509-3p on mRNA transcript abundance and YAP1 protein levels in HEYA8 and OVCAR8. (**A**) Relative expression of miR-509-3p by Taq-man q-PCR after transient transfection. Times (hr) are after transfection with a miR mimic. (**C, D**) Relative expression levels for genes from the 11-gene invasive signature or that are EMT-related transcription factors. (C) HEYA8 cells, (D) OVCAR8 cells.(**E, F**) Relative expression levels for predicted targets that are associated with migration and invasion. Results are for 3 biological replicates, each with 3 technical replicates, 72 hr after transfecting a miR-509-3p mimic. Nontreated cells (NT) were the control. NC = negative control using a scrambled miR mimic. Error bars represent standard errors. *, ** and *** indicate $P < 0.1$, 0.01, and 0.001. (**G**) YAP1 protein levels by Western blot after miR-509-3p mimic treatment, at 72 h, 96 h and 120 h after transfection in HEYA8 and OVCAR8 cell lines. (**H**) YAP1 protein levels by Western blot after miR-509-3p mimic, 509-3p inhibitor and siYAP1 treatment, at 72 h after transfection in HEYA8 and OVCAR8 cell lines.

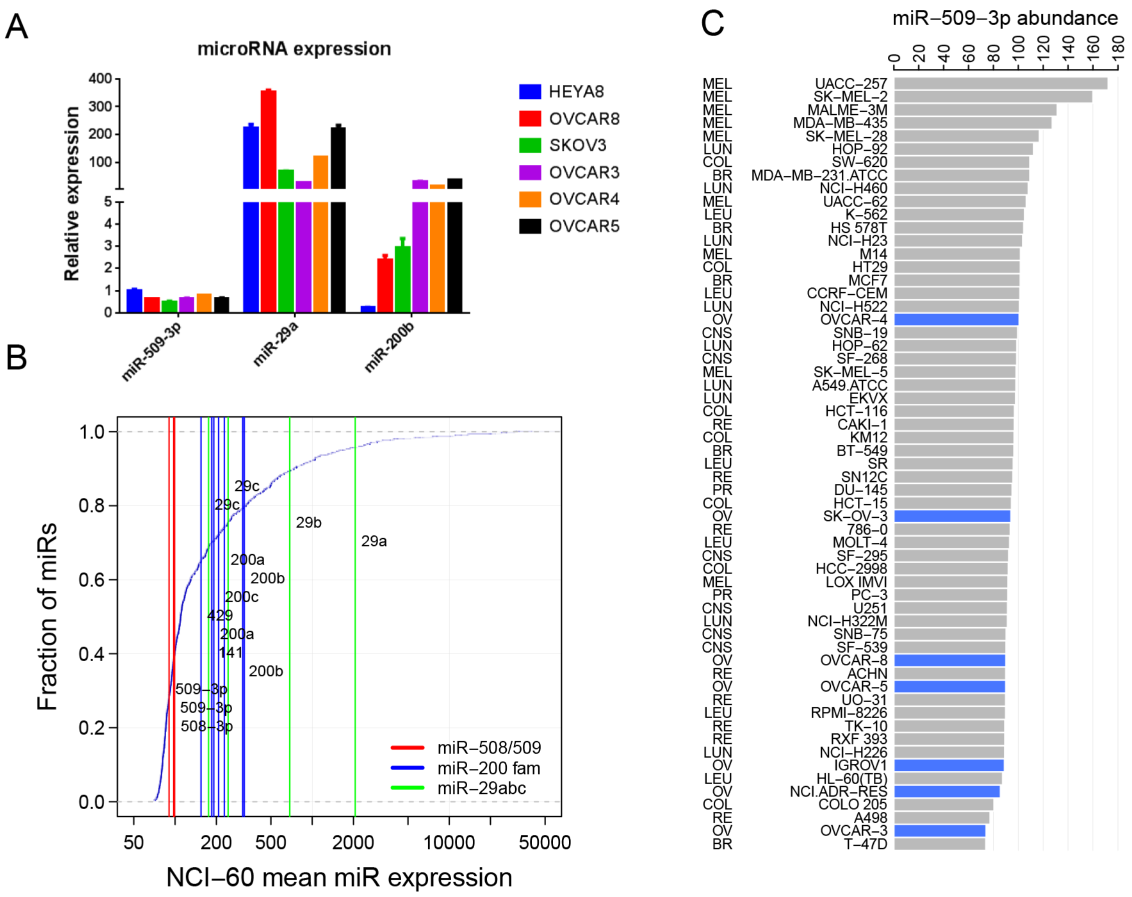

Supplementary Figure S4: miR-509-3p is expressed at relatively low levels. (**A**) Relative expression of miR-509-3p, miR-29a and miR-200b by qPCR in the six ovarian cancer cell lines, normalized to 509-3p expression in HEYA8. RNU48 expression was used as endogenous control. Results are for 3 biological replicates, each with 3 technical replicates. (**B**) Distribution function of 1050 miRs of NCI-60 cell lines microarray data in the GEO: GSE26375 data matrix, with vertical lines showing mean expression values for a subset of miRs. The table gives mean log2 expression and mean expression for miR-508-3p, miR-509-3p (GSE26375 IDs 11141 and 21499), miR-200 family, and miR-29abc. (**C**) Distribution of expression of miR-509-3p (ID 11141) across the NCI-60 cell lines, with blue bars showing the ovarian cancer (OV) cell lines.

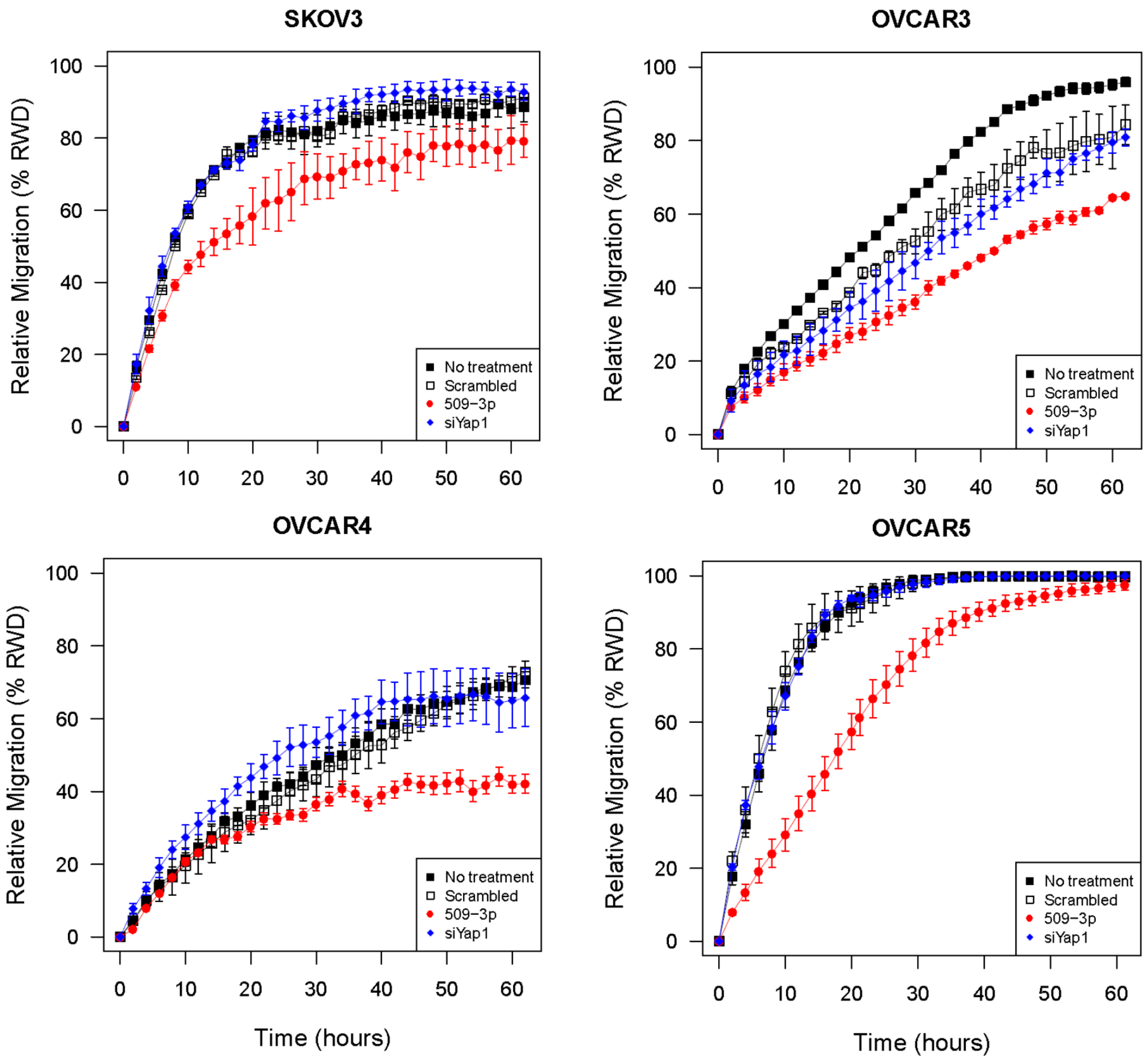

Supplementary Figure S5: Effects of miR-509-3p and siRNA to YAP1 on migration in four ovarian cancer cell lines.

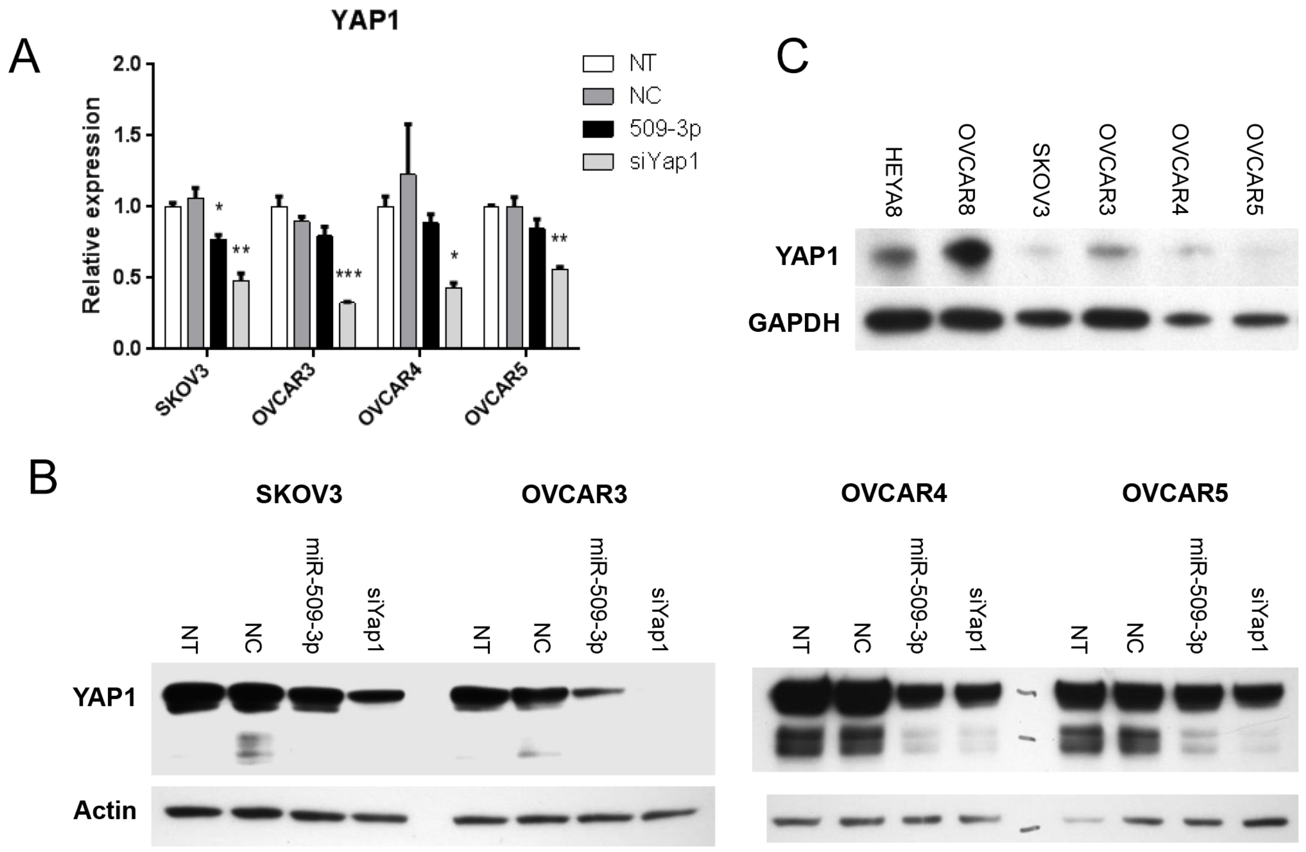

Supplementary Figure S6: YAP1 mRNA transcript abundance and protein levels in miR-509-3p mimic treated/nontreated cells. (**A**) Relative mRNA transcript levels by qPCR at 72 h after miR-509-3p and siYAP1 transfection. Error bars represent standard deviations. *, **, and *** indicate $P < 0.1$, 0.01 and 0.001. (**B**) YAP1 protein levels by Western blot after miR-509-3p mimic and siYAP1 treatment, at 72 h after transfection. (**C**) YAP1 protein levels in six ovarian cancer cell lines by Western blot.

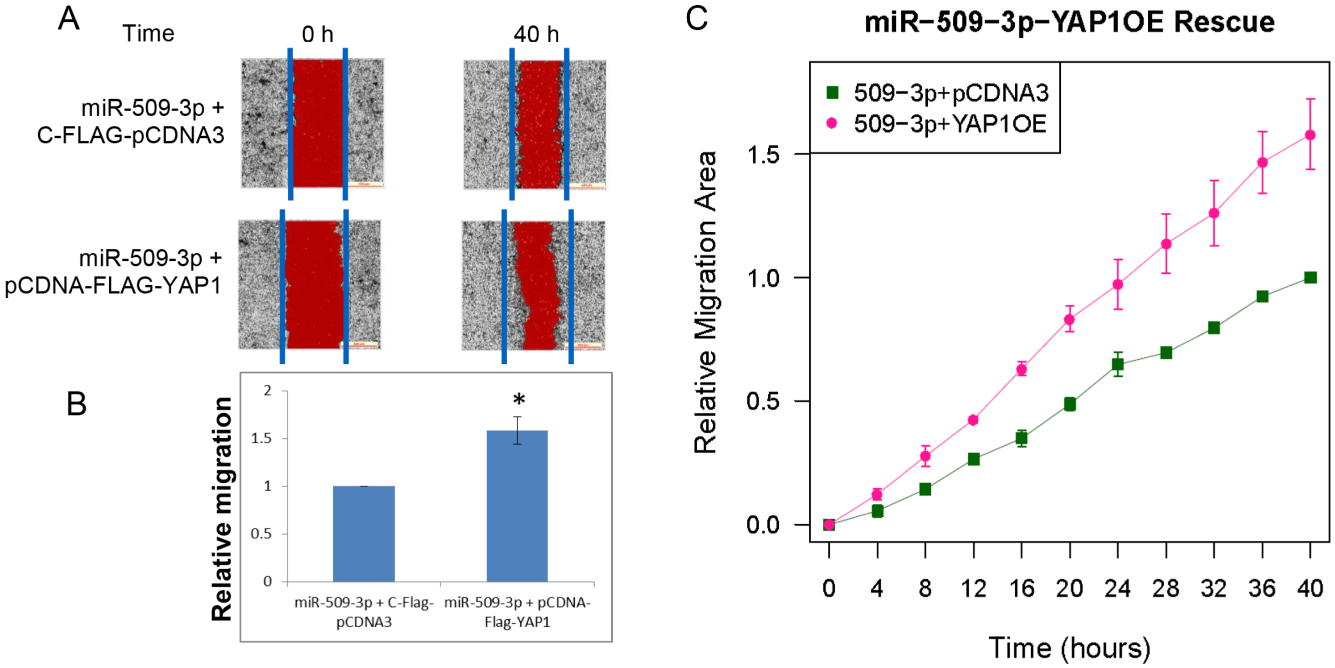

Supplementary Figure S7: Exogenous YAP1 rescues the inhibitory effects of miR-509-3p on migration in OVCAR8 cells. Cells were transfected with both an miR-509-3p mimic, and either exogenous pCDNA Flag-Yap1 or an empty vector C-FLAG-pCDNA3. (**A**) Representative micrographs of wound healing experiments at 0 and 40 hours. Lines show the widths of the initial scratches. (**B**) The bar graph shows the relative migration of cell transfected with miR-509-3p + YAP1 overexpression comparing to the migration of cell transfected with miR-509-3p and empty vector at 40 h. $P < 0.05$. (**C**) Wound-healing time-course. Results are for 3 biological replicates.

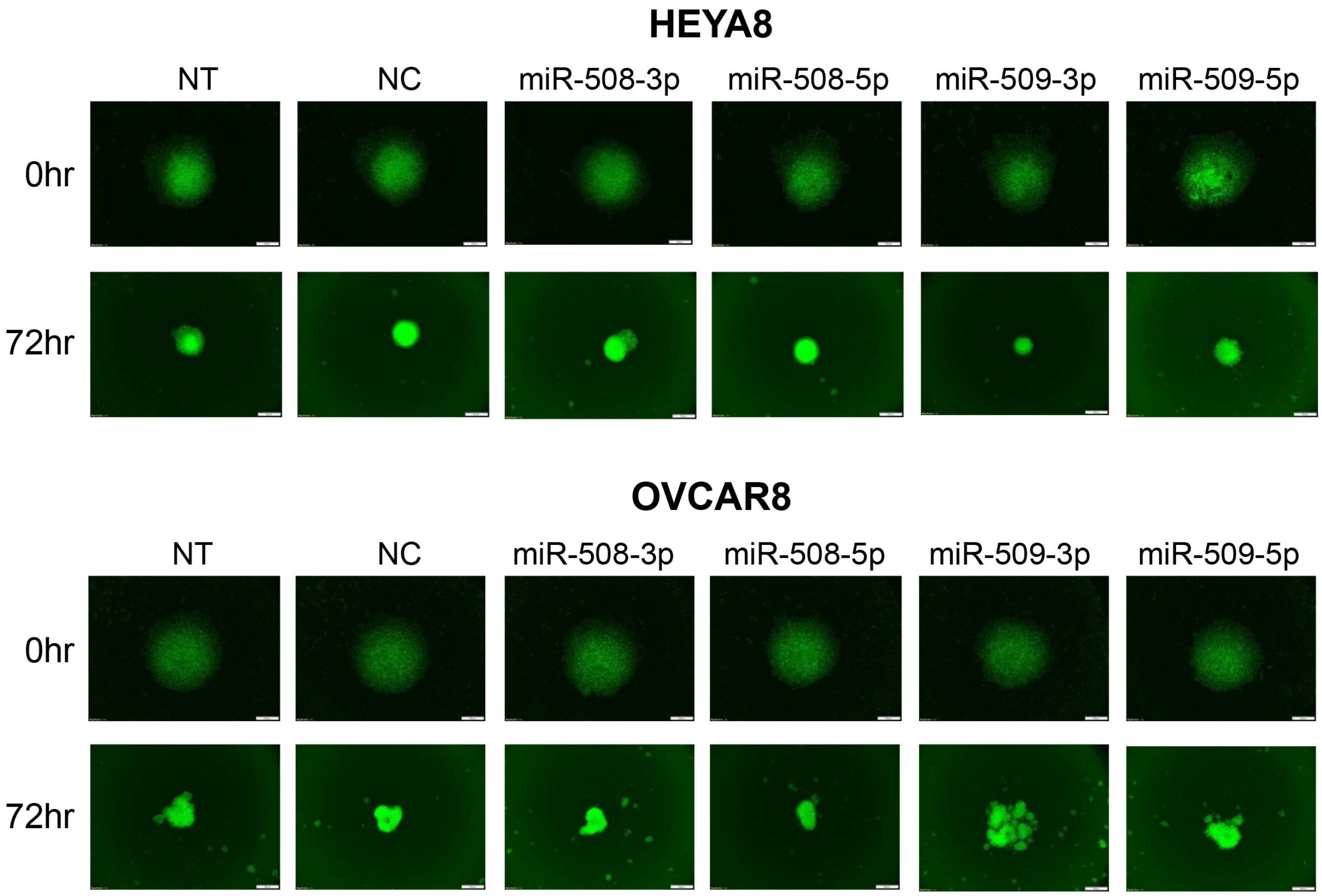

Supplementary Figure S8: Effects of miR-508-3p, 508-5p, 509-3p and 509-5p on spheroid formation in HEYA8 and OVCAR8 cells.
